# IL-27 shapes the immune properties of human astrocytes and their impact on encountered human T lymphocytes

**DOI:** 10.1186/s12974-022-02572-1

**Published:** 2022-09-01

**Authors:** Florent Lemaître, Negar Farzam-kia, Ana Carmena Moratalla, Yves Carpentier Solorio, Marie-Laure Clenet, Olivier Tastet, Aurélie Cleret-Buhot, Jean Victor Guimond, Elie Haddad, Pierre Duquette, J. Marc Girard, Alexandre Prat, Catherine Larochelle, Nathalie Arbour

**Affiliations:** 1 Department of Neurosciences, Université de Montréal and Centre de Recherche du CHUM (CRCHUM), 900 St-Denis Street, Room R09.464, Montreal, QC H2X 0A9 Canada; 2 Centre de Recherche du Centre Hospitalier de L’Université de Montréal (CRCHUM), Montreal, QC Canada; 3 CLSC Des Faubourgs, CIUSSS du Centre-Sud-de-L’Ile-de-Montréal, Montréal, QC Canada; 4 Department of Microbiology, Infectious Diseases, and Immunology and Department of Pediatrics, Centre de Recherche du Centre Hospitalier, Université de Montréal, Universitaire Sainte-Justine (CHU Sainte-Justine), Montreal, QC Canada; 5 MS-CHUM Clinic, 900 St-Denis Street, Montreal, QC H2X 0A9 Canada

**Keywords:** Cytokines, Glial cells, T lymphocytes, Transcription factors, T cell motility

## Abstract

**Background:**

Interleukin-27 (IL-27) can trigger both pro- and anti-inflammatory responses. This cytokine is elevated in the central nervous system (CNS) of multiple sclerosis (MS) patients, but how it influences neuroinflammatory processes remains unclear. As astrocytes express the receptor for IL-27, we sought to determine how these glial cells respond to this cytokine and whether such exposure alters their interactions with infiltrating activated T lymphocytes. To determine whether inflammation shapes the impact of IL-27, we compared the effects of this cytokine in non-inflamed and inflamed conditions induced by an IL-1β exposure.

**Main body:**

Transcriptomic analysis of IL-27-exposed human astrocytes showed an upregulation of multiple immune genes. Human astrocytes increased the secretion of chemokines (CXCL9, CXCL10, and CXCL11) and the surface expression of proteins (PD-L1, HLA-E, and ICAM-1) following IL-27 exposure. To assess whether exposure of astrocytes to IL-27 influences the profile of activated T lymphocytes infiltrating the CNS, we used an astrocyte/T lymphocyte co-culture model. Activated human CD4^+^ or CD8^+^ T lymphocytes were co-cultured with astrocytes that have been either untreated or pre-exposed to IL‑27 or IL-1β. After 24 h, we analyzed T lymphocytes by flow cytometry for transcription factors and immune molecules. The contact with IL-27-exposed astrocytes increased the percentages of T-bet, Eomes, CD95, IL-18Rα, ICAM-1, and PD-L1 expressing CD4^+^ and CD8^+^ T lymphocytes and reduced the proportion of CXCR3-positive CD8^+^ T lymphocytes. Human CD8^+^ T lymphocytes co-cultured with human IL-27-treated astrocytes exhibited higher motility than when in contact with untreated astrocytes. These results suggested a preponderance of kinapse-like over synapse-like interactions between CD8^+^ T lymphocytes and IL-27-treated astrocytes. Finally, CD8^+^ T lymphocytes from MS patients showed higher motility in contact with IL-27-exposed astrocytes compared to healthy donors’ cells.

**Conclusion:**

Our results establish that IL-27 alters the immune functions of human astrocytes and shapes the profile and motility of encountered T lymphocytes, especially CD8^+^ T lymphocytes from MS patients.

**Supplementary Information:**

The online version contains supplementary material available at 10.1186/s12974-022-02572-1.

## Background

Multiple sclerosis (MS) is an inflammatory disease of the central nervous system (CNS) characterized by axon demyelination, neuronal loss, and glial cell activation [1, 2]. Increasing evidence underlines the critical role of astrocytes in the development of MS lesions [3]. Reactive astrocytes in MS tissues exhibit functional modifications and produce pro- and anti-inflammatory mediators, including cytokines, chemokines, adhesion molecules, and immunomodulatory molecules [3–5]. Moreover, astrocytes can modulate the properties of both neural cells and infiltrating leukocytes due to their localization within the CNS. We have recently shown that IL-1β-inflamed astrocytes increase the motility of activated human CD8^+^ T lymphocytes using an in vitro co-culture live imaging system [6]. The mediators altering the capacity of astrocytes to shape the properties of CNS infiltrating T lymphocytes remain incompletely resolved.

IL-27 is part of the IL-6/IL-12 cytokine family and is composed of p28 and Epstein–Barr virus-induced gene 3 (EBI-3) subunits. Two chains form the IL-27 receptor (IL-27R): IL-27Rα and gp130, the latter being shared with other cytokines [7–9]. IL-27 can exhibit pro- and anti-inflammatory properties depending on the physiological and pathological contexts [10]. IL-27 can act on naïve CD4^+^ and CD8^+^ T lymphocytes to promote the expression of T-bet, which is involved in Th1/Tc1 cell differentiation and the production of IFN-γ and granzyme B [11–13]. In contrast, this cytokine can dampen the production of cytokines by established Th1 or Th17 lymphocytes or reduce the expression of GATA-3 and RORγt. These two transcription factors are, respectively, associated with Th2 and Th17 differentiation [14, 15].

We and others have demonstrated that IL-27 expression is elevated in the brain and cerebrospinal fluid of MS patients [16, 17]. We have shown that astrocytes express IL-27R in MS lesions and could thus respond to these elevated levels. Moreover, we have established that primary cultures of human astrocytes respond to IL-27 by inducing STAT1 phosphorylation but not STAT3, in contrast to what is observed in leukocytes [16]. In addition, human astrocytes pre-exposed to inflammatory cytokines exhibit an enhanced STAT1 response to IL-27 compared with untreated cells [16]. The mechanisms whereby IL-27 shapes astrocytes’ properties under resting and inflammatory conditions remain incompletely resolved. Notably, increasing evidence suggests that IL-27 can modulate MS and experimental autoimmune encephalomyelitis (EAE) course [18–20], but whether astrocytes contribute to such effects is still unclear.

In this study, we investigated the response of human astrocytes to IL-27 under non-inflamed and inflamed conditions. We induced in vitro inflammatory conditions by exposing astrocytes to IL-1β. Indeed, this inflammatory mediator is upregulated in MS, but also in brain injury, neurodegenerative diseases, and infections [21–23]. Using a microarray approach, we found that IL-27 triggered the expression of multiple immune-related genes in astrocytes. We confirmed that IL‑27 induced the secretion of chemokines and enhanced the protein expression of several immune molecules. We assessed whether exposure to IL-27 modified the capacity of human astrocytes to influence the immune profile of encountered activated human CD4^+^ and CD8^+^ T lymphocytes. We report that IL-27-treated astrocytes increased the proportion of T lymphocytes expressing the transcriptional factors T-bet and EOMES as well as immune molecules known to regulate T cell activity and migration. We found that IL-27-treated astrocytes altered the motility of CD8^+^ T lymphocytes from healthy donors and MS patients. Our results establish that astrocytes’ response to IL-27 changes their immune properties and their capacity to shape the profile of infiltrating activated T lymphocytes.

## Material and methods

### Ethics approval and consent to participate

These studies were approved by the Centre Hospitalier de l’Université de Montréal (CHUM) ethics boards (BH07.001, HD07.002). Fetal (17–21 weeks) brain tissue was obtained after written informed consent (ethical committee of CHU Sainte-Justine, Montreal QC, Canada, CER #2126; University of Washington Birth Defects Research Laboratory Seattle, Washington, USA, STUDY00000380). All healthy controls (HC) and MS patients gave written informed consent for blood donation in accordance with the local ethical committee, and studies were approved by the CHUM ethical boards (BH 07.001 and HD 07.002). Patients were diagnosed with relapsing–remitting MS (RRMS) by highly trained MS-neurologists (PD, JMG, AP, CL) according to the revised 2017 McDonald criteria [24] and were recruited from the CHUM MS-Clinic.

### Isolation and culture of human astrocytes

Fetal brain tissues were processed to isolate human astrocytes using a well-established protocol [6]. Briefly, CNS tissues were mechanically and enzymatically digested, and dissociated neural cells were plated in complete DMEM with 5% (v/v) fetal bovine serum (FBS) and antibiotics. Highly enriched astrocytes from passages 4 or 5 (> 95%) [6] were used for all experiments.

### Microarray

Astrocytes were left either untreated or were inflamed with recombinant human IL-1β (R&D Systems distributed by Cedarlane, 20 ng/mL) for 2 days. Then, recombinant human IL-27 (R&D Systems distributed by Cedarlane, 100 ng/mL) was added or not to untreated and IL-1β-inflamed astrocytes for 24 h. Cells were washed with PBS and lysed in Trizol. RNA samples were purified using RNeasy plus mini kit according to the manufacturer’s protocol (Qiagen). All samples had RNA integrity number (RINs) > 9.7. Transcriptome-wide analysis of gene expression was performed by Genome Québec using the Clariom S HT array profiling more than 20,000 well-annotated genes. Differential expression analysis was done using the statistical software R. Intensity files were processed using the oligo package v1.54.1 and normalized with the RMA method. Differential expression was assessed with the limma package v3.41.6. Genes were identified as significantly differentially expressed when the adjusted *p*-value < 0.05 and the absolute fold-change > 1.3. Gene Set Enrichment Analysis (GSEA) was performed using the fgsea package in R. This approach ranks all genes according to their t-statistic, taking both the directionality and the significance of the statistical test into account. Microarray data have been deposited in the NCBI-GEO repository with the accession number GSE201555 (private access token for reviewers: erojmoyopnqzraj).

### Supernatant and ELISA

Astrocytes, either untreated or pre-inflamed with recombinant human IL-1β (20 ng/mL) for 48 h, were exposed or not to recombinant human IL-27 (100 ng/mL), and then after 24 h, supernatants were harvested and stored at − 20 °C. CXCL9, CXCL10, IL-18BP, and IL-27 were assessed using ELISA kits (R&D Systems distributed by Cedarlane Laboratories). CXCL11 levels were assessed using U-Plex immunoassay kit according to the manufacturer’s instructions (Meso Scale Discovery).

### CD4^+^ and CD8^+^ T cell isolation, activation, and labeling

Peripheral blood mononuclear cells (PBMC) were isolated from healthy donors’ and MS patients’ blood collected in EDTA-coated tubes using Ficoll density gradient as routinely performed [25, 26]. CD8^+^ T lymphocytes were first isolated using CD8 Microbeads (Miltenyi Biotec), while the negative fraction devoid of CD8^+^ T lymphocytes was used to isolate CD4^+^ T lymphocytes using CD4 Microbeads (Miltenyi Biotec) according to the manufacturer’s instructions. Purity was > 95% for both T cell subsets; a representative flow cytometry analysis of isolated CD4 and CD8 T lymphocytes is shown in Additional file 1: Fig. S1. CD4^+^ and CD8^+^ T lymphocytes cultured in complete Iscove medium (containing FBS 10%, sodium pyruvate 1 mM, L-glutamine 2 mM, MEM nonessential amino acids 1%, β-mercapto-ethanol 1 µM, and antibiotics) were activated for 5 days on plate-bound anti-CD3 (OKT3 clone, eBioscience-Life technology, 2.5 μg/mL for CD4^+^ T cells and 5 μg/mL for CD8^+^ T cells) in the presence of soluble anti-CD28 antibody (BD Biosciences, 1 µg/mL). After five days, activated CD4^+^ or CD8^+^ T lymphocytes were harvested for co-culture experiments.

### Astrocyte–T cell co-culture assay

Astrocytes (2 × 10^5^ cells/well in 24 well plates) were either untreated or treated with recombinant human IL-27 (100 ng/mL) or IL-1β (20 ng/mL) for 24 h. Subsequently, astrocytes were washed with fresh medium before adding 1 million activated CD4^+^ or CD8^+^ T lymphocytes (T cell: astrocyte ratio, 5:1); astrocyte–T cell co-cultures were incubated for 24 h at 37 °C prior flow cytometry analysis. In some experiments, soluble IL-27Rα (500 ng/mL) was added 1 h before adding T lymphocytes onto astrocytes.

### Flow cytometry

Astrocytes and T lymphocytes were stained for surface and/or intracellular molecules as previously described [16, 25]. Briefly, astrocytes and astrocyte–T cell co-cultures were detached using PBS containing 5 mM EDTA. To exclude dead cells, cells were stained with LIVE/DEAD Fixable Aqua Dead Cell Stain (Molecular Probes™, ThermoFisher Scientific) in PBS for 30 min at 4 °C. Cells were blocked with normal mouse immunoglobulins (mIgG) (6 ug mIgG/million of cells) (Invitrogen) for 15 min prior to being incubated with fluorochrome-labeled antibodies targeting surface antigen (see Table 1) for 30 min at 4 °C. To assess the expression of transcriptional factors, cells were fixed and permeabilized using FOXP3/Transcription factor staining buffer set (eBioscience-Life technology) according to the manufacturer instructions and subsequently incubated with fluorochrome-labeled antibodies targeting transcription factors. Cells were acquired on a BD LSRII flow cytometer (BD Biosciences) and analyzed using FlowJo software (Treestar). Doublets were excluded using SSC and FSC Height and Width as recommended by the Flow Cytometry Network (www.thefcn.org); single events were first gated on the SSC-H vs. SSC-W and then on the FSC-H vs. FSC-W dot plots. Appropriate isotype controls were used in all steps to confirm staining specificity. The change in median fluorescence intensity (MFI) was calculated by subtracting the fluorescence of the isotype from that of the stain.

**Table 1:** Antibodies for flow cytometry analysis

Targeted human antigen–fluorochrome	Clone	Concentration used per stain	Vendor
CD4–BV786	SK3	1 µg/mL	BD Biosciences
CD8–Super Bright 702	RPA-T8	0.5 µg/mL	eBioscience-Invitrogen
CD54 (ICAM-1)–BV421	HA58	0.5 µg/mL	BD Biosciences
CD95 (Fas)–FITC	DX2	1.25 µg/mL	BD Biosciences
CXCR3–BV421	G025H7	20 µg/mL	BioLegend
PD-1 PE	J105	5 µg/mL	eBioscience-Invitrogen
PD-L1–biotin	MIH1	5 µg/mL	eBioscience-Invitrogen
HLA-ABC Pacific Blue	W6/32	40 µg/mL	BioLegend
HLA-E–PE	3D12	10 µg/mL	ThermoFisher
IL-18Rα–biotin	H44	10 µg/mL	BioLegend
T-bet–BV421	O4-46	1 µg/mL	BD Biosciences
EOMES–PE-Cy7	WD1928	0.48 µg/mL	ThermoFisher
RORγt–Alexa Fluor 647	Q21-559	0.25 µg/mL	BD Biosciences
GATA-3–PE-cf594	L50-823	0.060 µg/mL	BD Biosciences
Streptavidin–BV605		2ug/mL	BD Biosciences

### Live imaging of astrocyte–T cell co-culture

As previously published, human astrocytes were stained with Orange CMRA and plated in µ-dish 35 mm Quad [6]. CD8^+^ T lymphocytes were collected after the 5-day activation and stained with CFSE before being added to untreated, or IL-27 treated astrocytes in a 4:1 ratio (CD8 T cell:astrocyte). CD8^+^ T lymphocytes obtained from untreated MS patients and age/sex-matched healthy donors were co-cultured with astrocytes from the same donor. Astrocyte–T cell co-cultures were imaged using a spinning disc confocal microscope for 2 h (1 frame/min) and analyzed as previously published [6]. The arrest coefficient of each cell was calculated as the proportion of time the cell’s speed was below 2 μm/min.

### Statistics

Data analysis was performed using Prism 9.2 (GraphPad) or R software. When data passed the D’Agostino and Pearson omnibus normality test or the Shapiro–Wilk normality test when the sample size was too small to perform the former, t-test or one-way ANOVA test followed by Fisher’s LSD test was used. When data did not pass the normality test, the Wilcoxon or the Friedman test followed by a Dunn’s multiple comparison test was used to compare paired groups. Statistical tests used are indicated in figure legends. Values were considered statistically significant when probability (*P*) values were equal or below 0.05 (*), 0.01 (**), 0.001 (***), or 0.0001 (****).

## Results

### Human astrocytes exposed to IL-27 show differential expression of genes associated with immune functions

**Fig. 1:**
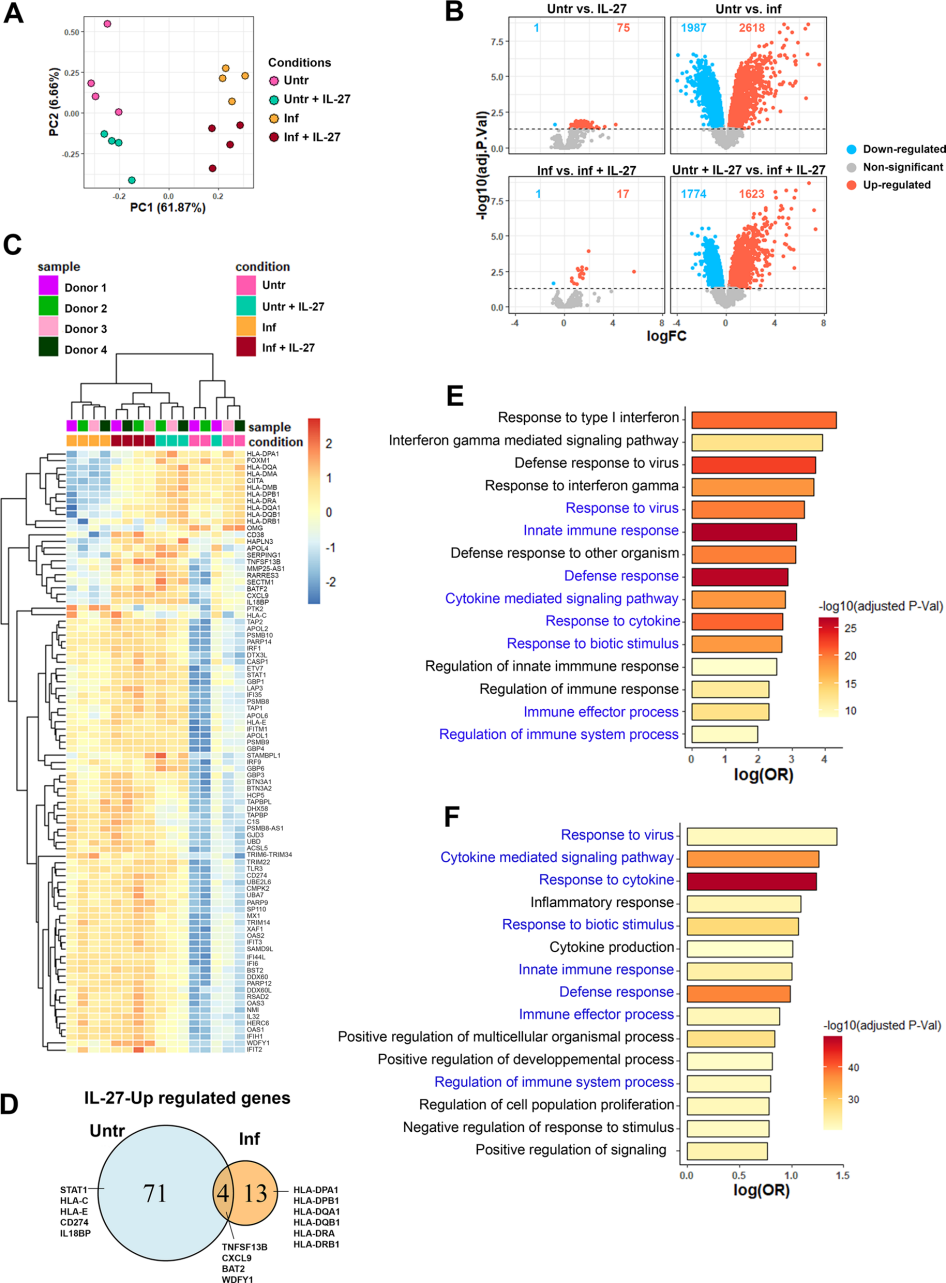
Human astrocytes exposed to IL-27 and/or IL-1β show differential gene expression associated with immune functions. Gene expression profiles of human astrocytes either untreated (Untr) or IL-1β-inflamed (Inf) and then exposed or not to IL-27 (Untr + IL-27; and Inf + IL-27) for 24 h were analyzed. *n* = 4 individual donors. **A** Principal component analysis (PCA) of the transcriptomic profile of Untr (pink), Untr + IL-27 (green), Inf (yellow), and Inf + IL-27 (brown). **B** Volcano plots showing the number of upregulated (red) and downregulated (blue) genes in Untr. vs. IL-27-exposed, untreated vs. inflamed (IL-1β-exposed), inflamed (IL-1β-exposed) vs. inflamed + IL-27 astrocytes, and untreated-IL-27 vs. IL-1β + IL-27. **C** Heatmap of the upregulated genes induced by IL-27 in both non-inflamed and inflamed astrocytes. DEGs and samples are ordered according to hierarchical clustering within the space of the displayed DEGs (pheatmap package). Each individual donor is color-coded (donors 1 to 4). **D** Venn diagram presenting the number of IL-27-up-regulated genes in either untreated (Untr) or inflamed (Inf) astrocytes or shared by both conditions. Examples of genes are indicated. **E**, **F** Gene Set Enrichment Analysis (GSEA) of the upregulated genes induced by IL-27 **E** or IL-1β. **F** Exposed astrocytes. Genes were ranked according to their t-statistic for the corresponding comparisons. The log_2_ of the odd ratio (logOR) is presented on the x-axis and the –log_10_ of the adjusted P value (-log10(adjusted P-val)) is represented with the color legend. GSEA shared between IL-27 and IL-1β are shown in blue

We have previously demonstrated that human astrocytes respond to IL-27 by triggering STAT1 phosphorylation and that pre-treatment with inflammatory cytokines can potentiate such response [16]. To determine the broad impact of IL-27 on human astrocyte gene expression patterns, we assessed 20 800 annotated genes using a microarray approach. Primary cultures of human astrocytes were either left untreated or pre-inflamed with IL-1β for 48 h before being exposed to IL-27. The inflammation induced by IL-1β triggered numerous transcriptional changes compared with untreated astrocytes (Fig. 1A, B). Principal component analysis (PCA) showed 61.87% of the variance (PC1) across astrocyte samples was associated with inflammation. On PC2, we observed that 6.66% of the variance was associated with astrocytes exposition to IL-27 (Fig. 1A). IL-27 induced significantly differentially expressed genes (DEGs) in both untreated and pre-inflamed astrocytes; most DEGs were upregulated and more abundant in non-inflamed astrocytes compared to inflamed astrocytes (75 and 17, respectively) (Fig. 1B). IL-1β triggered 2618 upregulated, and 1987 downregulated DEGs in astrocytes compared with untreated cells (Fig. 1B). The inflamed (IL-1β) + IL-27 condition induced a higher number of up (1623) or downregulated (1774) DEGs in astrocytes compared with the IL-27 condition.

Multiple genes upregulated in response to IL-27 were also enhanced by IL-1β treatment in astrocytes (Fig. 1C). IL-27 upregulated four genes in both untreated and inflamed astrocytes (Fig. 1D). To investigate the biological pathways involved in the astrocytes’ responses to IL-27, we performed a Gene Set Enrichment Analysis (GSEA) using the *fgsea* package in R (Fig. 1E). Enriched pathways were mainly associated with immune-related processes and included type I interferon responses, and innate immune responses. Enriched pathways triggered by IL-1β (Fig. 1F) were linked to similar pathways to those induced by IL-27 (indicated in blue; e.g., response to virus, cytokine mediated pathway, response to cytokine) but also included different pathways (identified in black; e.g., inflammatory response, positive regulation of multicellular organismal process). Our results establish that IL-27 alters the immune transcriptomic profile of human astrocytes.

### IL-27 enhances the secretion and surface expression of several immune molecules by human astrocytes

**Fig. 2:**
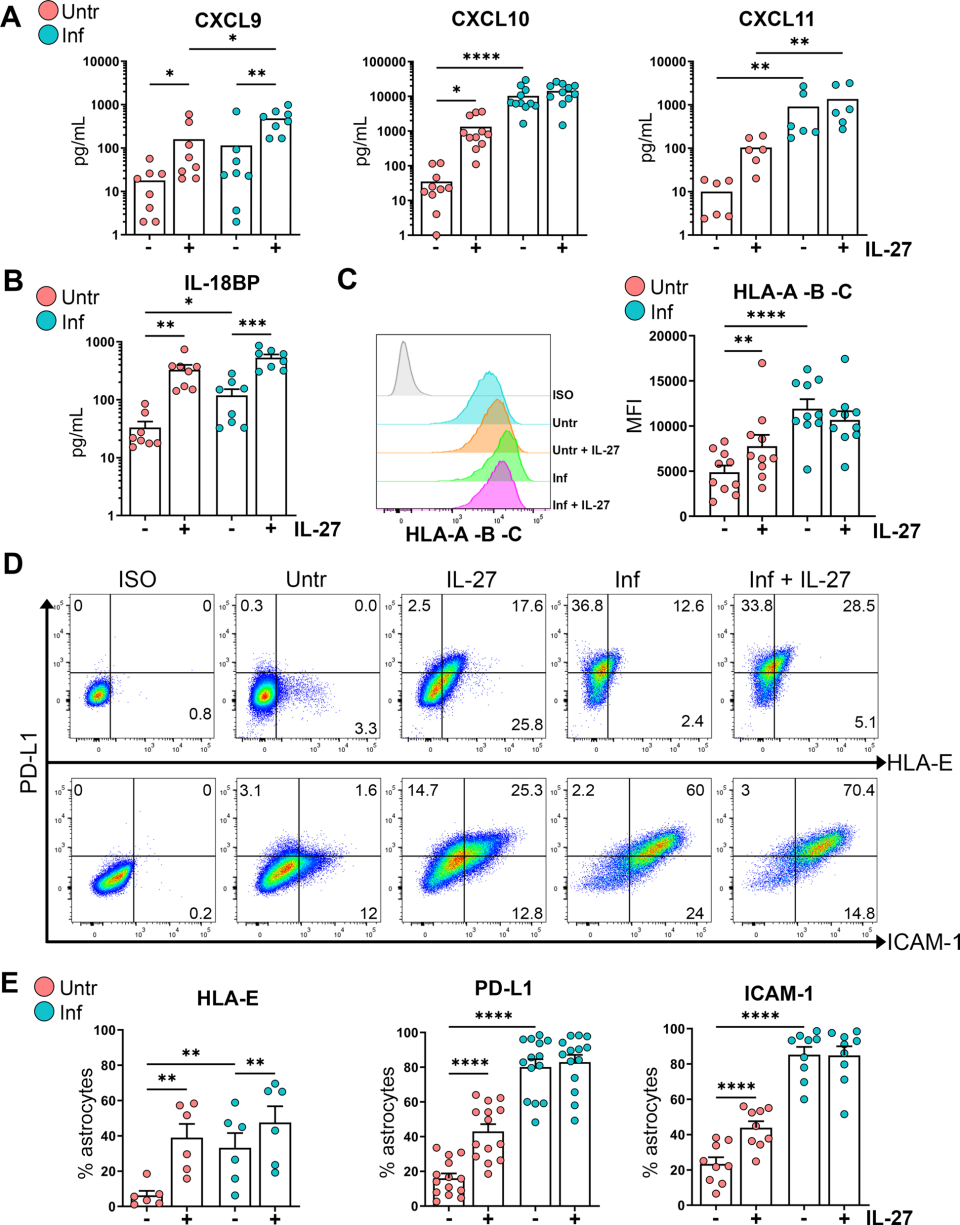
IL-27 enhances the secretion and surface expression of several immune molecules by untreated or IL-1β-exposed human astrocytes. Untreated (untr) and IL-1β-treated astrocytes (inf) were exposed to IL-27 (100 ng/mL) for 24 h. Supernatants were tested for the secretion of **A** CXCL9, CXCL10, CXCL11, and **B** IL-18BP by ELISA. **C**–**E** Astrocytes were analyzed for surface expression of HLA-ABC, HLA-E, PD-L1, and ICAM-1 using flow cytometry. **C** Representative histogram of HLA-ABC detection and geometric mean fluorescence intensity are shown. **D** Representative dot plots for expression of HLA-E, PD-L1, and ICAM-1. **E** Percentage of positive astrocytes for HLA-E, PD-L1, and ICAM-1. **A**–**E** Each dot represents one donor. Paired Friedman test (**A**) or one‐way paired ANOVA followed by Fisher's LSD test. **B**, **C**, **E** comparing Untr and Inf astrocytes in the presence vs. absence of IL‐27 from each group. *n* = 6–14, *P < 0.05, **P < 0.01, ***P < 0.001 and ****P < 0.0001

Among IL-27-upregulated genes, we selected a subset of immune molecules for validation at the protein level by ELISA or flow cytometry. The chemokine CXCL9, which belongs to the same chemokine family as CXCL10 and CXCL11, was significantly upregulated by IL-27 at the transcriptional level (Fig. 1D). These chemokines were assessed in supernatants of non-inflamed and inflamed astrocytes exposed to IL-27. IL-27 triggered a significant increase in CXCL9 and CXCL10 secreted levels by non-inflamed astrocytes, and a similar trend was observed for CXCL11 (Fig. 2A). While IL-27 further increased CXCL9 levels in IL-1β pretreated astrocytes, CXCL10 and CXCL11 secretion remained unchanged. Astrocytes exposed to IL-27 expressed elevated levels of mRNA encoding for IL-18 binding protein (IL-18BP), an inhibitor of the pro-inflammatory cytokine IL-18 (Fig. 1D). IL-27 significantly increased the secreted levels of IL-18BP in both non-inflamed and inflamed astrocytes compared to their non-exposed counterparts (Fig. 2B).

IL-27 upregulated genes encoding for major histocompatibility complex (MHC) class I and II molecules in astrocytes (Fig. 1C, D). Using flow cytometry, we detected elevated HLA-ABC (MHC class I molecules) expression by non-inflamed astrocytes exposed to IL-27 compared to untreated cells (Fig. 2C). However, IL-27 did not alter HLA-ABC expression levels on IL-1β-inflamed astrocytes. HLA-E, a non-classical MHC class Ib molecule, was expressed by a more significant proportion of astrocytes upon exposure to IL-27, regardless of whether astrocytes were previously inflamed or not (Fig. 2D, E). Our microarray analysis also identified PD-L1 (CD274) among upregulated genes (Fig. 1C, D); we confirmed that a higher percentage of non-inflamed astrocytes expressed this molecule upon IL-27 exposure compared to untreated cells (Fig. 2D, E). IL-1β-inflamed astrocytes exhibited a very high proportion of PD-L1-positive cells, which was not further enhanced by IL-27 (Fig. 2D, E). We previously reported that IL-27 enhances the expression of the adhesion molecule ICAM-1 by human T lymphocytes [25]. Similarly, IL-27 significantly increased the proportion of ICAM-1-positive astrocytes compared to untreated cells (Fig. 2D, E). Over 80% of IL-1β-inflamed astrocytes expressed ICAM-1, and IL-27 did not increase this percentage (Fig. 2D, E). Our results establish that exposing human astrocytes to IL-27 increases the protein expression of several immune molecules that could be implicated in chemotaxis, antigen presentation, cell adhesion, and modulation of T cell activity.

### IL-27-treated astrocytes favor the expression of the T-bet and EOMES transcription factors by activated human CD4^+^ and CD8^+^ T lymphocytes

**Fig. 3:**
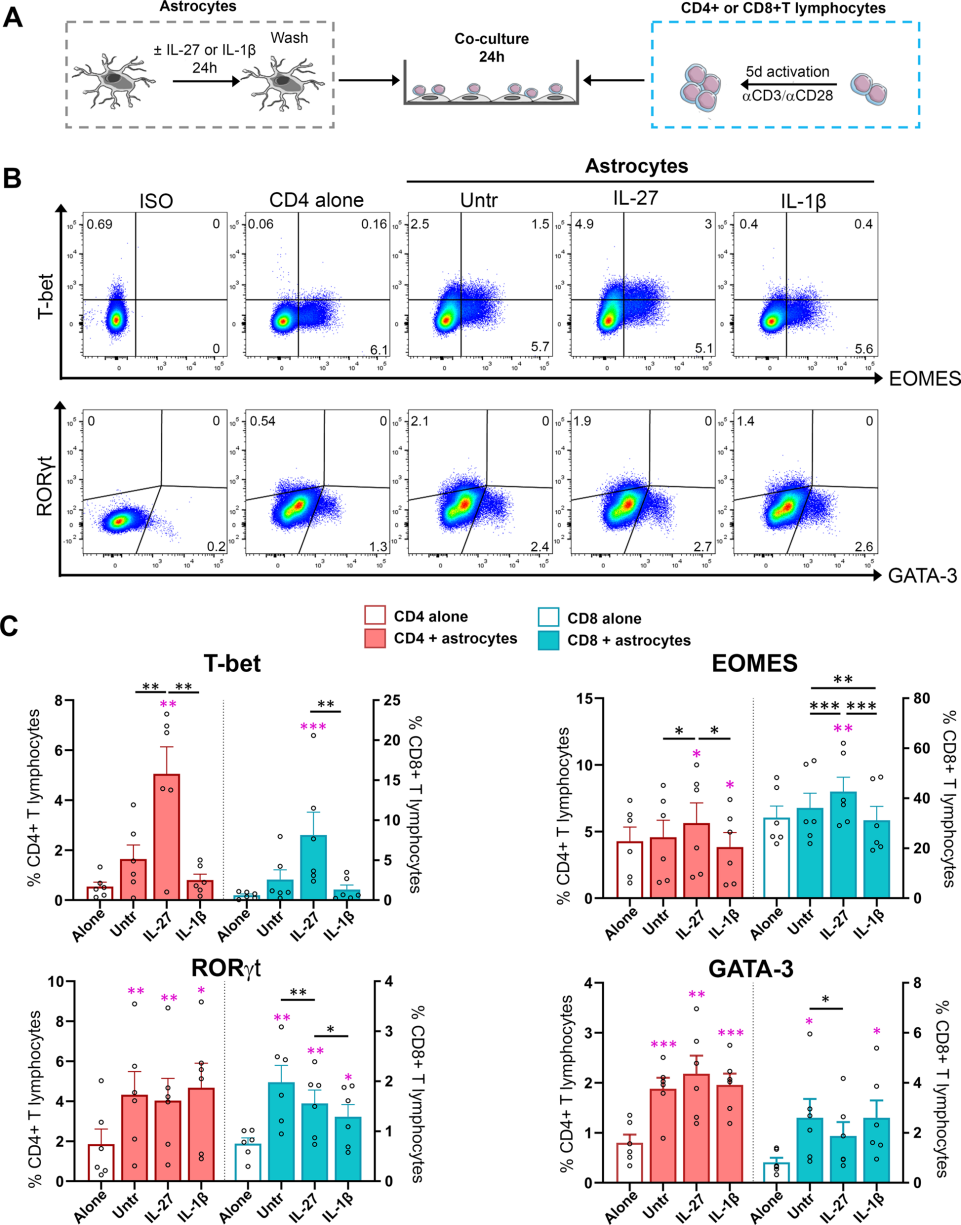
IL-27-treated astrocytes enhance expression of T-bet and EOMES by co-cultured CD4^+^ and CD8^+^ T lymphocytes. **A** Experimental procedure for T cell–astrocyte co-cultures. Human astrocytes were treated or not with IL-27 or IL-1β for 24 h. αCD3/αCD28 activated human CD4^+^ or CD8^+^ T lymphocytes were added to washed human astrocytes and co-cultured for 24 h prior to being collected for flow cytometry analysis. **B** Representative dot plots of CD4^+^ T lymphocytes alone or co-cultured with untreated, IL-27 or IL-1β-treated astrocytes for detection of T-bet, EOMES, RORγt, and GATA-3. **C** Percentage of positive CD4^+^ (red, left axis) and CD8^+^ T lymphocytes (blue, right axis) expressing T-bet, EOMES, RORγt and GATA-3 are shown as mean ± SEM; each dot represents one donor. Paired Friedman test (T-bet for CD8^+^ T lymphocytes) or one‐way ANOVA followed by Fisher's LSD test (for all other comparisons) were used. P values comparing T lymphocytes alone (empty bars) and T cells co-cultured with untreated or treated astrocytes are shown as pink stars. P values for comparison within T lymphocytes–astrocytes co-cultured groups are shown as black stars above lines. *n* = 6; *P < 0.05, **P < 0.01, ***P < 0.001 and ***P < 0.0001

To investigate whether exposure of astrocytes to IL-27 influences the immune profile of CNS infiltrating T lymphocytes, we established an astrocyte/T lymphocyte co-culture model (Fig. 3A). To mimic peripheral activation, isolated human CD4^+^ or CD8^+^ T lymphocytes were activated with anti-CD3 and anti-CD28 antibodies. In parallel, astrocytes were exposed or not with IL-27 or IL-1β and then carefully washed before adding activated T lymphocytes. After 24 h of co-culture, we assessed the expression of transcriptional factors associated with Th1/Tc1, Th17/Tc17, and Th2/Tc2 cell subsets using flow cytometry. IL-27-treated astrocytes increased the proportion of CD4^+^ and CD8^+^ T lymphocytes expressing the transcription factors T-bet and/or EOMES (Fig. 3B, C), whereas IL-1β-treated astrocytes did not significantly alter such expression compared to untreated astrocytes (Fig. 3B, C). A substantially greater proportion of CD4^+^ T lymphocytes co-cultured with astrocytes expressed the transcription factors RORγt or GATA-3 compared to CD4^+^ T lymphocytes not in contact with astrocytes regardless of astrocyte treatments (Fig. 3C). The percentage of CD8^+^ T lymphocytes expressing RORγt or GATA-3 also significantly increased upon contact with astrocytes (Fig. 3C). However, co-culture with IL-27-treated astrocytes reduced the percentage of RORγt or GATA-3 expressing CD8^+^ T lymphocytes compared to untreated astrocytes. Upon co-culture with IL-1β-inflamed astrocytes, a lower proportion of CD8^+^ T lymphocytes expressed RORγt compared to untreated or IL-27-treated astrocytes conditions. Our results show that human astrocytes can modify the expression of transcriptional factors associated with specific activation profiles/polarizations of T lymphocytes. Moreover, IL-27-exposed astrocytes exhibited a distinct impact than IL-1β exposed astrocytes in the capacity to shape the expression of T-bet and EOMES by T lymphocytes.

### T lymphocytes specifically change the surface expression of immune molecules in contact with IL-27-treated astrocytes.

**Fig. 4:**
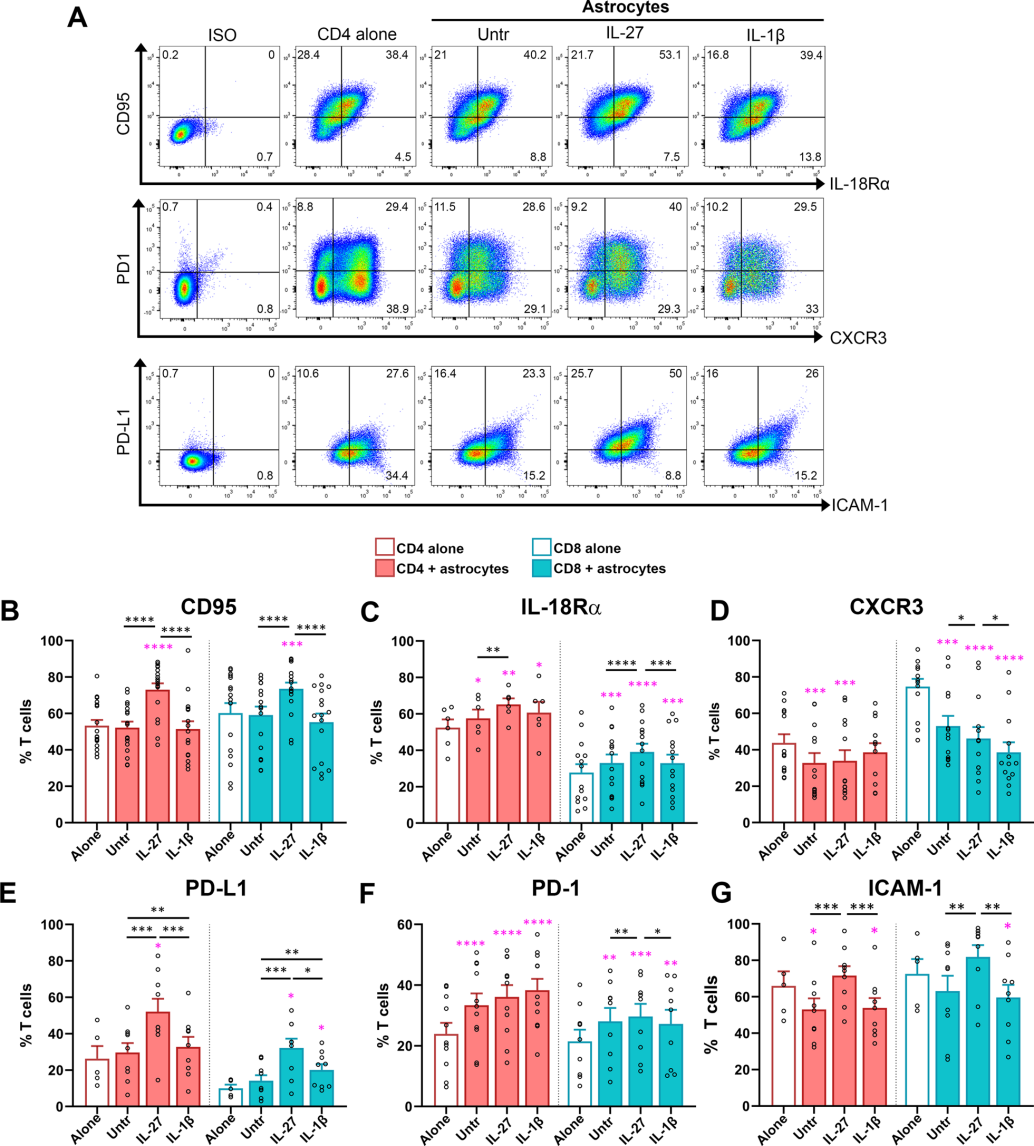
T lymphocytes modify their expression of surface immune molecules upon contact with IL-27-treated astrocytes. CD4^+^ and CD8^+^ T lymphocytes alone or co-cultured with untreated, IL-27 or IL-1β-treated astrocytes for 24 h were analyzed by flow cytometry. **A** Representative dot plots of CD4^+^ T lymphocytes in each condition for the detection of CD95, IL-18Rα, CXCR3, PD-1, PD-L1, and ICAM-1. **B**–**G** Percentages of positive CD4^+^ (red) and CD8^+^ T lymphocytes (blue) expressing. **B** CD95, **C** IL-18Rα, **D** CXCR3, **E** PD-L1, **F** PD-1, and **G** ICAM-1 are shown as mean ± SEM. Each dot represents one donor. Paired One‐way ANOVA followed by Fisher's LSD test for comparison between conditions. P values comparing T lymphocytes alone (empty bars) and T cells co-cultured with untreated or treated astrocytes are shown as pink stars. P values for comparison within T lymphocyte–astrocyte co-culture groups are shown as black stars. *n* = 6–17; *P < 0.05, **P < 0.01, ***P < 0.001 and ***P < 0.0001

We sought to determine whether T lymphocytes encountering astrocytes modify their surface expression of markers implicated in immune functions. CD95 (also known as Fas) is involved not only in the induction of apoptosis, but also T cell activation [27, 28]. While untreated and IL-1β-treated astrocytes did not affect the proportion of CD4^+^ and CD8^+^ T lymphocytes expressing CD95, IL-27-treated astrocytes significantly boosted these proportions (Fig. 4A, B). Since the IL-18 pathway was modified in IL-27-treated astrocytes (Figs. 1D, 2B), we investigated the expression of the IL-18Rα on T lymphocytes. An elevated proportion of CD4^+^ and CD8^+^ T lymphocytes expressed IL-18Rα upon co-culture with astrocytes, and this augmentation was more prominent in the IL-27-treated astrocyte condition (Fig. 4A, C) for both subsets. As CXCR3 is the cognate receptor for CXCL9, CXCL10, and CXCL11, three chemokines abundantly secreted by astrocytes in response to IL-27 treatment (Fig. 2A), we evaluated its expression by CD4^+^ and CD8^+^ T lymphocytes. The proportion of CXCR3-expressing CD4^+^ T lymphocytes was reduced upon co-culture with untreated and IL-27-treated astrocytes compared to T cells left alone. IL-1β-treated astrocytes did not have such an impact (Fig. 4A, D). The proportion of CD8^+^ T lymphocytes expressing CXCR3 was decreased in the presence of astrocytes compared to T cells alone, regardless of treatment, but both IL-27 and IL-1β treatment potentiated this decrease (Fig. 4D). The proportion of PD-L1-expressing CD4^+^ T lymphocytes was not altered upon co-culture with untreated and IL-1β-inflamed astrocytes; in contrast, the IL-27-pretreated astrocyte condition significantly increased such expression (Fig. 4E). Notably, the percentage of PD-L1 expressing CD8^+^ T lymphocytes was modestly elevated in the IL-1β-exposed astrocyte condition, but the IL-27-exposed astrocyte condition had a greater impact on PD-L1 expression (Fig. 4E). Moreover, PD-1 expression was also significantly increased in both CD4^+^ and CD8^+^ T lymphocytes in contact with astrocytes. IL-27-treated astrocytes had a higher impact on such expression by CD8^+^ T lymphocytes than other astrocyte conditions (Fig. 4 F). Finally, untreated and IL-1β-treated astrocytes decreased the proportion of CD4^+^ and CD8^+^ T lymphocytes expressing ICAM-1 compared to T cells alone (Fig. 4G). However, both T lymphocyte subsets co-cultured with IL-27-exposed astrocytes maintained a high level of ICAM-1.

We tested whether the IL-27 added to astrocytes could participate in the observed effects on T lymphocytes in our co-culture assay. We assessed the cytokine by ELISA in astrocyte cultures and observed around 100,000 pg/mL, corresponding to the dose used to treat astrocytes (Additional file 2: Fig. S2A). In the T cell:astrocyte co-cultures, we detected around 3.5 pg/mL demonstrating that more than 95% of the added IL-27 was removed by our extensive washes (Additional file 2: Fig. S2A). To investigate whether this residual IL-27 could mediate the observed effects, we performed IL-27 blocking experiments. We used the soluble human IL-27Rα, an antagonist of IL-27 [25, 29], that efficiently blocks the capacity of IL-27 to act on human T lymphocytes [25]. Astrocytes exposed to IL-27 for 24 h were extensively washed, and then IL-27Rα was added or not one hour at an excessive dose (500 ng/mL) before the addition of T lymphocytes (Additional file 2: Fig. S2B). The presence of soluble IL-27Rα did not abolish the capacity of IL-27-treated astrocytes to increase the expression of CD95, PD-L1, and ICAM-1 by co-cultured T lymphocytes (Additional file 2: Fig. S2C). The IL-27Rα blocking experiment supports the notion that soluble IL-27 is not necessary in the astrocyte:T cell co-cultures for the observed effects on T lymphocytes. Overall, our results support the notion that IL-27-exposed astrocytes shape the expression of several immune molecules in T lymphocytes.

### CD8^+^ T lymphocytes from MS patients exhibit higher motility than those from HC upon contact with IL-27-exposed astrocytes

**Fig. 5:**
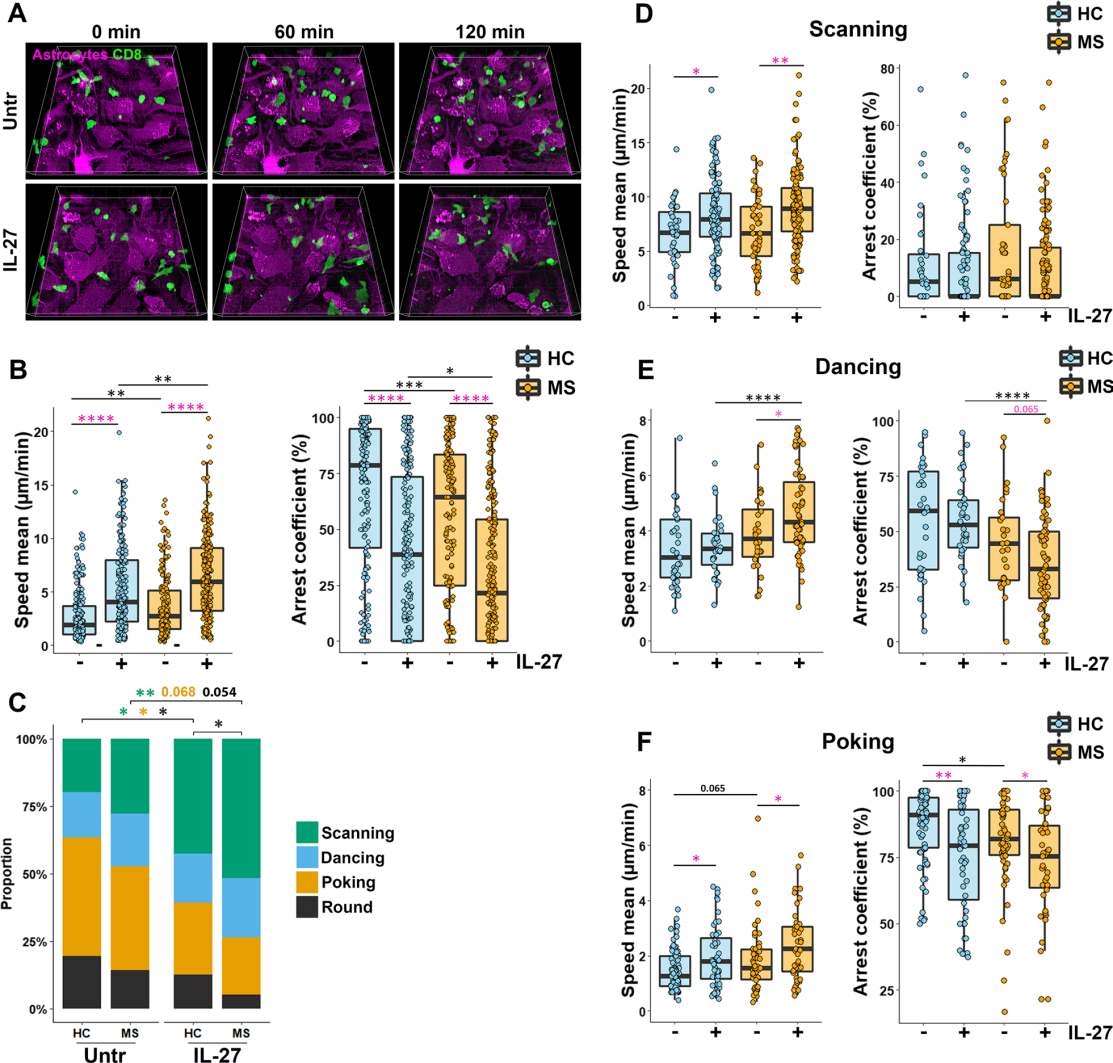
MS patients’ CD8^+^ T lymphocytes exhibit higher motility than HC’s upon contact with IL-27-exposed astrocytes. Orange CMRA-labeled astrocytes were treated or not with IL-27 for 24 h. αCD3/αCD28 activated human CD8^+^ T lymphocytes from healthy control (HC) and MS patient (MS) were added to washed astrocytes, and cells were imaged every minute for 2 h. **A** Three-dimensional time-lapse view at *T* = 0, *T* = 1 and *T* = 2 h of activated CD8^+^ T lymphocytes (green) from one MS donor co-cultured on untreated or IL-27-treated astrocytes (magenta). **B** Box plot presenting the speed mean (µm/min) and arrest coefficient (%) measured for each individual CD8^+^ T lymphocyte from HC (blue boxes) and MS patients (orange boxes) tracked on untreated or IL-27-treated astrocytes. The line represents the median in each box. Comparisons of CD8^+^ T lymphocytes from HC and MS patients (pink stars) and T lymphocytes from each group (black stars) are shown. **C** Proportions of CD8^+^ T lymphocytes from HC and MS patients exhibiting the scanning, dancing, poking, and round behaviors when co-cultured with untreated or IL-27-treated astrocytes. One‐way ANOVA followed by Fisher's LSD test for comparison between conditions. P values are color-coded for each behavior. **D**–**F** Box plots presenting the speed mean (µm/min) and the arrest coefficient (%) measured for **D** scanning, **E** dancing, **F** poking CD8^+^ T lymphocytes from HC (blue boxes) and MS patients (orange boxes) tracked on untreated or IL-27-treated astrocytes. The line represents the median in each box. **B**, **D**, **E**, **F** Statistical p values for comparisons of CD8^+^ T lymphocytes from HC and MS patients are shown as black stars, whereas comparisons of T lymphocytes within same donor group are shown as pink stars. Data for astrocyte–CD8^+^ T cell co-cultures are pooled from 5 distinct experiments, 382 cells from 6 HC and 380 cells from 6 MS patients. Wilcoxon or t-test was used. *P < 0.05, **P < 0.01, ***P < 0.001 and ***P < 0.0001, ****P < 0.00001

Using our recently published time-lapse co-culture model [6], we compared the motility of CD8^+^ T lymphocytes from healthy donors and MS patients in co-culture with untreated or IL-27-treated astrocytes (Fig. 5A, Additional files 3 and 4: movies 1–2). CD8^+^ T lymphocytes from healthy and MS donors exhibited a significantly higher mean speed and reduced arrest coefficient when encountering IL-27-exposed astrocytes compared to the untreated astrocytes (Fig. 5B). Moreover, CD8^+^ T lymphocytes from MS patients showed a significantly higher mean velocity and reduced arrest coefficient than T lymphocytes from healthy donors when co-cultured with untreated or IL-27-treated astrocytes (Fig. 5B), suggesting increased motility.

We categorized T cell behaviors employing our previously published classification: scanning and dancing, which present kinapse-like motility; poking and round, showing synapse-like motion [6]. The proportion of CD8^+^ T lymphocytes exhibiting a scanning behavior from MS patients and healthy donors was significantly increased upon co-culture with IL-27-pretreated astrocytes compared to untreated counterparts (Fig. 5C). Significantly less healthy donors’ CD8^+^ T lymphocytes showed a poking or round behavior upon contact with IL-27-treated astrocytes (Fig. 5C). Similar results were obtained for CD8^+^ T lymphocytes from MS patients without reaching statistical significance (Fig. 5C). However, the proportion of CD8^+^ T lymphocytes from MS patients harboring a round behavior upon contact with IL-27-treated astrocytes was significantly reduced compared with cells from healthy donors (Fig. 5C). These results suggest that CD8^+^ T lymphocytes from both MS patients and healthy donors preferentially adopted kinapse-like characteristics (scanning and dancing behaviors) upon contact with IL-27-exposed astrocytes.

We sought to determine whether IL-27-exposed astrocytes affect the motility of specific behavior subsets. Scanning CD8^+^ T lymphocytes from MS patients and healthy donors exhibited a significantly greater average speed when contacting IL-27-pretreated astrocytes than those encountering untreated astrocytes (Fig. 5D). However, scanning cells from MS patients and healthy donors presented a similar arrest coefficient on both untreated and IL-27-treated astrocytes. IL-27-treated astrocytes did not significantly impact the speed and arrest coefficient of dancing CD8^+^ T lymphocytes from healthy donors (Fig. 5E). However, dancing CD8^+^ T lymphocytes from MS patients showed elevated speed mean and reduced arrest coefficient when in contact with IL-27-treated astrocytes compared with those on untreated astrocytes (Fig. 5E). Moreover, dancing CD8^+^ T lymphocytes from MS patients in the IL-27-treated astrocyte condition presented a significantly higher average speed and reduced arrest coefficient compared with healthy donor counterparts (Fig. 5E). Poking CD8^+^ T lymphocytes from both donors’ groups presented an enhanced mean speed correlating with a reduced arrest coefficient on IL-27-treated astrocytes compared with counterparts in contact with untreated astrocytes (Fig. 5F). Finally, mean speed and arrest coefficient of round CD8^+^ T lymphocytes, which were less abundant, were not affected by IL-27 treatment of astrocytes (data not shown). Taken together, our data suggest that CD8^+^ T lymphocytes from MS patients exhibited greater motility on untreated and IL-27 treated astrocytes than those from healthy controls.

## Discussion

In the current study, we demonstrated that IL-27, which is elevated in the CNS of MS patients [16, 17, 25], induces the production of immune molecules by non-inflamed and inflamed human astrocytes (Figs. 1 and 2). We established that IL-27-exposed astrocytes shape the profiles of activated human CD4^+^ and CD8^+^ T lymphocytes, including their expression of transcriptional factors and surface immune molecules (Figs. 3 and 4). Finally, we demonstrated that, upon contact with human astrocytes, CD8^+^ T lymphocytes from MS patients exhibit greater motility than lymphocytes from healthy controls; IL-27-pre-exposed astrocytes further increased CD8^+^ T lymphocytes’ motility (Fig. 5).

IL-27 upregulated the expression of multiple immune molecules in human astrocytes, although this cytokine had more impact on non-inflamed than IL-1β-inflamed cells (Figs. 1, 2). Only four DEGs were commonly upregulated by IL-27 in both conditions, including CXCL9, whose secretion was increased upon adding IL-27 (Fig. 2). IL-1β induced numerous DEGs in astrocytes (Fig. 1), similarly to what has been published by others, using iPSC-derived astrocytes [30]. Numerous publications have underlined the extensive capacity of astrocytes to modify their properties in response to their environment [31, 32]. Notably, astrocytes can exhibit innate immune memory (also called trained innate immunity) such that their immune responses are shaped by a previous stimulation [32]. Our current and previous work [16] underline that IL-1β and other inflammatory cytokines, abundantly present in MS brain tissues, can influence the astrocytes’ response to IL-27. Indeed, we have previously shown that pre-treatment with IL-1β + TNF significantly increased the IL-27-induced STAT1 signaling in human astrocytes [16]. We can speculate that in vivo, human astrocytes are most likely exposed to multiple factors modulating their responses throughout their lifespan.

At the protein level, IL-27 increased the expression of CXCL9, CXCL10, CXCL11, IL-18BP, HLA-ABC, HLA-E, PD-L1 and ICAM-1 in non-inflamed astrocytes. However, IL-27 did not alter the high levels of CXCL10, HLA-ABC, PD-L1, and ICAM-1 triggered by IL-1β treatment. Nevertheless, IL-27 enhanced the expression of CXCL9, IL-18BP, and HLA-E by IL-1β-pretreated astrocytes (Fig. 2). IL-27 and IL-1β upregulated the expression of the same immune molecules by astrocytes. Both increased the secretion of CXCL10 and IL-18BP as well as the surface expression of HLA-ABC, HLA-E, PD-L1 and ICAM-1 (Fig. 2). Notably, several molecules upregulated by IL-27 or IL-1β have been shown to be elevated in MS brains, including MHC class I molecules, ICAM-1, and PD-L1 on astrocytes [5, 33–36]. IL-27 increases PD-L1 expression by other cells including human T lymphocytes [25]. IL-27 exerts pro- and anti-inflammatory effects on human astrocytes; this cytokine enhanced the expression of multiple immune molecules, some favoring an inflammatory milieu (e.g., CXCL10, HLA-ABC, ICAM-1, etc.), but other having regulatory functions (e.g., IL-18BR, PD-L1). Differences have been documented between fetal and adult human astrocytes [34]. Nonetheless, similarly to astrocytes in postmortem adult brain tissues, fetal astrocytes express IL-27R and upregulate the same immune mediators [5, 33–36].

Multiple molecules produced by IL-27 in human astrocytes could contribute to the mechanisms leading to the altered properties of encountered activated CD4^+^ and CD8^+^ T lymphocytes (Figs. 3, 4). We can speculate that ligation of CXCL9, CXCL10, and CXCL11 to CXCR3, which is known to trigger its internalization [37, 38], contributes to the reduced CXCR3 surface expression we observed on T lymphocytes upon contact with astrocytes, which secrete these three chemokines (Fig. 2). Such CXCR3-decreased expression was especially detected for CD8^+^ T lymphocytes in IL-27 or IL-1β-treated astrocyte conditions (Fig. 4D). Notably, CXCL10-expressing astrocytes and CXCR3-expressing leukocytes are observed in MS lesions but not in control brains [39]. Moreover, CXCL9 and CXCL10 ligation to CXCR3 can drive IFNγ production via STAT1/STAT4 signaling, promoting a Th1 response [40]. Moreover, STAT1/STAT4 signaling also drives T-bet expression [41]. Therefore, we suggest that IL-27-exposed astrocytes via the secretion of CXCL9 and CXCL10 can induce T-bet in activated T lymphocytes and thus amplify the Th1/Tc1-like phenotypes. The expression of T-bet and EOMES in both CD4^+^ and CD8^+^ T lymphocyte subsets was elevated in the IL-27-treated astrocyte condition but not in untreated or IL-1β-treated conditions (Fig. 3C, D). Such observations imply that other mechanisms are likely implicated as IL-1β triggered elevated production of CXCL9 and CXCL10 by astrocytes, but IL-1β -treated astrocytes did not alter T-bet nor EOMES expression by encountered T lymphocytes (Fig. 3). An elevated proportion of activated T lymphocytes expressed ICAM-1 and CD95 upon encountering IL-27-treated astrocytes, compared with untreated or IL-1β-treated astrocytes (Fig. 4). Ligation of CD95 to its ligand (FasL), also expressed on T lymphocytes, can lead to apoptotic and non-apoptotic effects depending on activation and context [42, 43]. Overall, our results suggest that IL-27-exposed astrocytes distinctly influence the profile (e.g., transcription factors, surface mediators) of encountered activated CD4^+^ and CD8^+^ T lymphocytes and favor Th1/Tc1-like phenotypes.

Increasing evidence supports the contribution of IL-18, a pro-inflammatory cytokine, to MS and EAE disease progression [44, 45]. Notably, signaling of IL-18 through IL-18Rα induces Th1 cells and IFN-γ production [46]. Elevated levels of IL-18Rα mRNA were reported in both CSF and PBMCs of MS patients [47, 48]. Moreover, IL-18Rα-deficient mice are resistant to EAE induction [47]. The elevated IL-18Rα expression by T lymphocytes upon contact with IL-27-exposed astrocytes could maintain Th1-like response within the CNS. However, IL-18BP, a secreted protein exhibiting a greater affinity to IL-18 than the cytokine’s cognate receptor (IL-18Rα), negatively regulates IL-18’s effects [45, 49]. We can speculate that elevated IL-18BPα levels secreted by astrocytes in response to IL-27 could have a beneficial impact on disease severity by abrogating IL-18 pro-inflammatory effects such as reducing Th1 response.

The PD-1/PD-L1 pathway negatively regulates T lymphocyte expansion, differentiation and activation [50]. In the absence of PD-L1, the development of EAE is more severe [51, 52]. PD-L1 and/or PD-1 expression by T lymphocytes can limit their activation, proliferation, and reduce Th1/Tc1 polarization [53, 54]. Our results support that IL-27 promotes the PD-1/PD-L1 pathway in human astrocyte–T lymphocyte interactions. While IL-27 upregulated PD-L1 expression by astrocytes, CD4^+^ and CD8^+^ T lymphocytes in contact with such IL-27-treated astrocytes also exhibited elevated proportions of PD-L1^+^ cells (Fig. 4). Our blocking experiment (Additional file 1: Fig. S2) showed that residual IL-27 in the co-culture was not essential for such PD-L1 upregulation by T lymphocytes.

Multiple factors can influence the motility of T lymphocytes as well as whether they form synapses or kinapses with other cells. LFA-1, expressed by T lymphocytes, and its ligand ICAM-1 can participate in kinapse formation and increase T cell motility [55–57]. Moreover, ICAM-1 has been implicated in MS and EAE pathogenesis [58–60]. Upon crossing the microvasculature, activated T lymphocytes infiltrating the CNS encounter the very abundant astrocytes. We showed that a greater proportion of IL-27-treated astrocytes expressed ICAM-1 than untreated cells (Fig. 2). Moreover, CD8^+^ T lymphocytes in contact with IL-27-exposed astrocytes exhibited a greater proportion of cells with kinapse-like motion than those on untreated astrocytes (Fig. 5). We can speculate that elevated expression of ICAM-1 combined with the secretion of CXCL9, CXCL10, and CXCL11 by IL-27-treated astrocytes could favor kinapse-like behavior by CD8^+^ T lymphocytes. We observed that CD8^+^ T lymphocytes from MS patients harbor higher motility than those from healthy donors, especially upon contact with astrocytes exposed to IL-27 (Fig. 5). Most CD8^+^ T lymphocytes within MS lesions have been shown to express higher levels of CD11a, one chain of the LFA-1 integrin [61]. Moreover, the motility of MOG-specific Th1 cells, which express elevated levels of LFA-1, infiltrating the subarachnoid space of the spinal cord during EAE was reduced by LFA-1 blockade [62]. The interaction between PD-1 on T lymphocytes, and PD-L1, on splenocytes, at the immunological synapse decreased T cell motility and favor synapse behavior in a viral infection mouse model [63]. Whether such PD-L1/PD-1 interaction could impact human T cell motility remains unclear. Whether highly motile T lymphocytes explore their microenvironment efficiently and consequently migrate beyond the perivascular astrocytes remains to be determined. Notably, CD8^+^ T lymphocytes are observed in the brain parenchyma of MS patients [64]. In animal models, different polarized T lymphocyte subsets exhibit different motility characteristics [65]. Further investigation will be necessary to elucidate the mechanisms shaping T cell motility upon contact with astrocytes and the impact on their tissue infiltration.

Additional investigations will be necessary to determine whether direct contact between astrocytes and T lymphocytes is necessary for the alterations we observed in T lymphocytes. For example, Boyden chambers could prevent direct contact between astrocytes and T lymphocytes. However, after the extensive washes, accumulated soluble factors are reduced and whether they continued to be released by astrocytes to affect T lymphocytes will need to be individually tested with blocking reagents as we did for IL-27 (Additional file 2: Fig. S2). Nonetheless, our live imaging data show that T lymphocytes established multiple sites of contact with astrocytes supporting dynamic interactions between these cell types.

## Conclusion

Finally, our study shows that astrocytes respond to IL-27 by modulating the expression of immune molecules involved in numerous biological processes. This response is different in non-inflamed compared with inflamed astrocytes. Astrocytes exposed to IL-27 shape the immune profile of activated CD4^+^ and CD8^+^ T lymphocytes in contact with these glial cells. Our work highlights the complex effect of IL-27 on very abundant glial cells and their subsequent encounter with infiltrating T lymphocytes. The elevated levels of IL-27 observed in MS brain tissues [16, 17] could act on both neural cells (e.g., astrocytes) and infiltrating T lymphocytes. These findings underline the importance of thoroughly characterizing the impact of IL-27 in the CNS of MS patients.

The role of astrocytes during the pathobiology of MS is multifaceted including the interactions between these abundant glial cells and infiltrating T lymphocytes [3, 66]. At earlier disease stages, other authors have suggested that astrocytes unravel multiple strategies to dampen neuroinflammatory processes [3, 66]. However, astrocytes participate in deleterious processes as the disease progresses by favoring the infiltration of leukocytes and their reactivation within the CNS. An improved understanding of those beneficial and deleterious effects could eventually be harnessed to develop novel therapeutic approaches [3, 66].

## Supplementary Information

**Additional file 1. Supplementary Figure 1:** Purity of isolated CD4 and CD8 T lymphocytes. T lymphocyte subsets were isolated using CD8 and CD4 Microbeads according to the manufacturer's instructions. Purity was routinely >95% as checked by flow cytometry.

**Additional file 2. Supplementary Figure 2:** IL-27-treated astrocytes enhance the expression of CD95, PD-L1 and ICAM-1 by T lymphocytes independently of residual IL-27 in the co-culture. **A** IL-27 levels measured in supernatants from untreated (white bar) or IL-27- exposed astrocytes (gray bar) and from astrocyte:CD4 T cell (pink) or astrocyte: CD8 T cell (green) co-cultures with astrocytes pretreated or not with IL-27. Each dot represents one sample. **B** Experimental procedure for T cell-astrocyte co-cultures. Human astrocytes were treated or not with IL-27 for 24h. Astrocytes were washed and then sIL-27Rα was added or not. One hour later, αCD3/αCD28 activated human CD4+ or CD8+ T lymphocytes were added to human astrocytes and co-cultured for 24h prior to being collected for flow cytometry analysis. **C** Percentage of positive T lymphocytes expressing CD95, PD-L1 and ICAM-1. n=3, 2 CD4+ T cell samples (white dots) and one CD8+ T cell sample (black dot).

**Additional file 3. Supplementary Video 1:** Three dimensional time lapse spinning disc microscopy view of activated CD8 T Lymphocytes (green) co-cultured with untreated astrocytes (magenta) over 2h. Gridlines: 20 μm, one picture per min, 20 frame/s.

**Additional file 4. Supplementary Video 2:** Three dimensional time lapse spinning disc microscopy view of activated CD8 T Lymphocytes (green) co-cultured with IL-27-treated astrocytes (magenta) over 2h. Gridlines: 20 μm, one picture per min, 20 frame/s.

## Data Availability

The datasets, including flow cytometry data used and analyzed during the current study, are available from the corresponding author upon reasonable request. Microarray data have been deposited in the NCBI-GEO repository with the accession number GSE201555 (private access token for reviewers: erojmoyopnqzraj).
