# Supplementary figures and images for: IL-27 shapes the immune properties of human astrocytes and their impact on encountered human T lymphocytes
Source: J Neuroinflammation. 2022 Sep 1;19:212. doi: 10.1186/s12974-022-02572-1 (PMC9434874; doi:10.1186/s12974-022-02572-1)

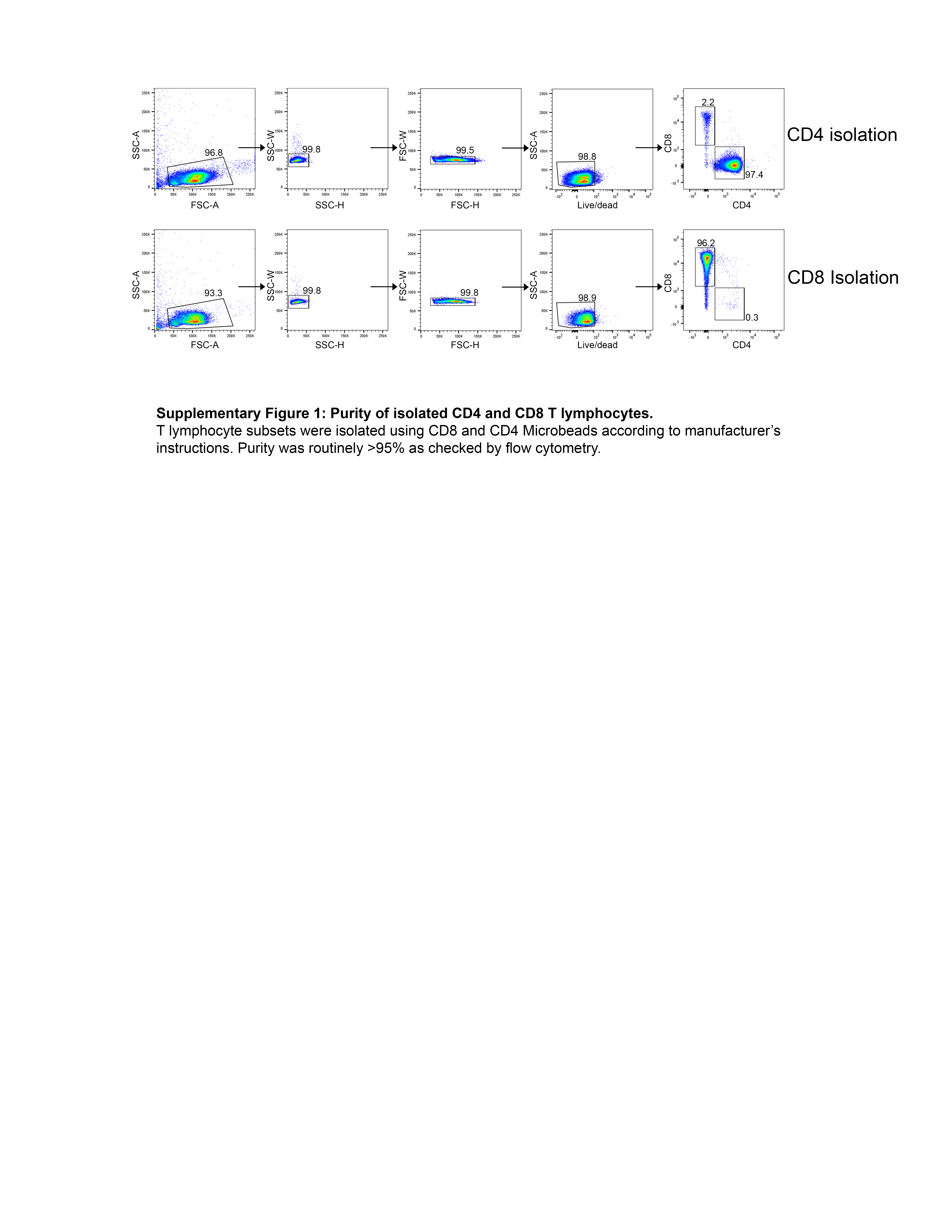

Supplement: Supplementary file 1 — Additional file 1. Supplementary Figure 1: Purity of isolated CD4 and CD8 T lymphocytes. T lymphocyte subsets were isolated using CD8 and CD4 Microbeads according to the manufacturer's instructions. Purity was routinely >95% as checked by flow cytometry. [file 12974_2022_2572_MOESM1_ESM.tif]

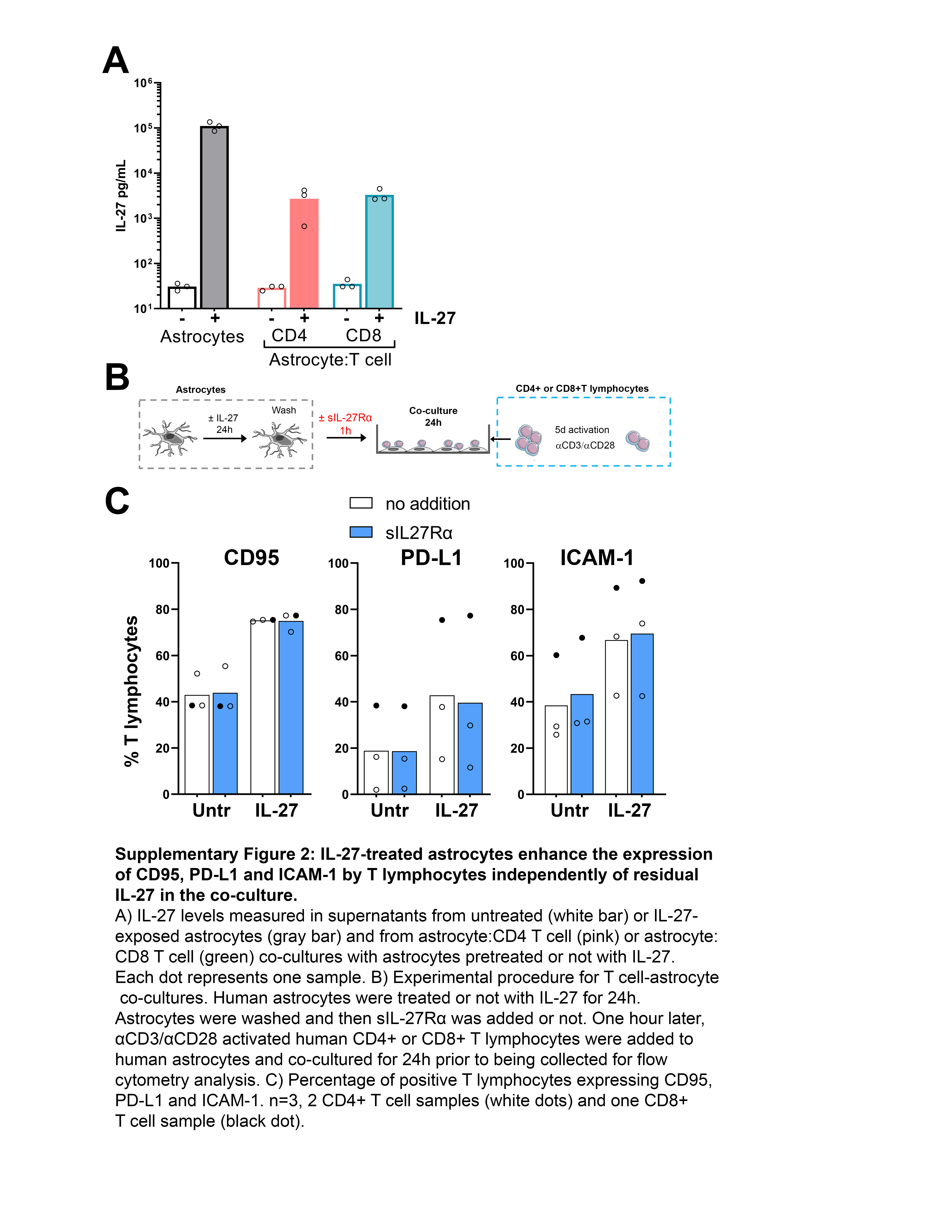

Supplement: Supplementary file 2 — Additional file 2. Supplementary Figure 2: IL-27-treated astrocytes enhance the expression of CD95, PD-L1 and ICAM-1 by T lymphocytes independently of residual IL-27 in the co-culture. A IL-27 levels measured in supernatants from untreated (white bar) or IL-27- exposed astrocytes (gray bar) and from astrocyte:CD4 T cell (pink) or astrocyte: CD8 T cell (green) co-cultures with astrocytes pretreated or not with IL-27. Each dot represents one sample. B Experimental procedure for T cell-astrocyte co-cultures. Human astrocytes were treated or not with IL-27 for 24h. Astrocytes were washed and then sIL-27Rα was added or not. One hour later, αCD3/αCD28 activated human CD4+ or CD8+ T lymphocytes were added to human astrocytes and co-cultured for 24h prior to being collected for flow cytometry analysis. C Percentage of positive T lymphocytes expressing CD95, PD-L1 and ICAM-1. n=3, 2 CD4+ T cell samples (white dots) and one CD8+ T cell sample (black dot). [file 12974_2022_2572_MOESM2_ESM.tif]
